# What Determines Levels of Mitochondrial Genetic Diversity in Birds?

**DOI:** 10.1093/gbe/evad064

**Published:** 2023-04-25

**Authors:** Alice Clark, Gizem Koc, Ying Eyre-Walker, Adam Eyre-Walker

**Affiliations:** School of Life Sciences, University of Sussex, Brighton, United Kingdom; School of Life Sciences, University of Sussex, Brighton, United Kingdom; School of Life Sciences, University of Sussex, Brighton, United Kingdom; School of Life Sciences, University of Sussex, Brighton, United Kingdom

**Keywords:** mitochondrial DNA, genetic diversity, Lewontin’s paradox, bird diversity

## Abstract

What determines levels of genetic diversity in mitochondrial DNA remains unresolved. We have investigated the factors that are correlated to the level of synonymous diversity of mitochondrial DNA in more than 300 bird species. We find that diversity is significantly correlated to clutch and range size, but not significantly correlated to many other variables including body mass, latitude, and longevity. The correlation between diversity and range appears to be a consequence of a correlation between range and effective population size since a measure of the effectiveness of natural selection, which is expected to be correlated to the effective population size, is also correlated to range. The slope of the relationship between diversity and range is shallow, consistent with Lewontin's paradox, and very similar to the relationship found in mammals.

**Significance**

Understanding why genetic variation varies between species is one of the central problems of population genetics. Here, we investigate what factors might influence genetic diversity in birds in mitochondrial DNA. We find that diversity is correlated to clutch and range size. The correlation with range is consistent with the expected influence of effective population size on levels of diversity. However, the relationship between diversity and range is shallow and weak.

## Introduction

The reason why genetic diversity at the DNA sequence level varies between species remains unclear despite more than 50 years of research (Leffler, et al. 2012; Charlesworth and Jensen 2022). The level of diversity is expected to depend upon a number of factors, principally the mutation rate, selection, and the effective population size.

Variation in the mutation rate appears to explain relatively little of the variation in nucleotide diversity between species for the nuclear genome, at least for multicellular eukaryotes, since mutation rates vary by approximately an order of magnitude (Lynch et al. 2016; Yoder and Tiley 2021), while levels of variation vary by at least two or three orders of magnitude (Leffler et al. 2012; Romiguier et al. 2014). For mitochondrial DNA, the situation is less clear. Early studies in mammals and birds suggested that mutation rate variation might be an important factor determining levels of diversity, because there is a correlation between diversity and the synonymous substitution rate (Nabholz et al. 2008, 2009). However, a subsequent analysis using phylogenetically independent pairs of species in mammals failed to find any evidence of a correlation between diversity and the synonymous divergence (James and Eyre-Walker 2020) suggesting that the mutation rate is probably not a major correlate of mitochondrial diversity, at least in mammals. Unfortunately, it is difficult to assess the role of mutation rate variation in determining levels of diversity in mitochondrial DNA because there are very few direct estimates of the mitochondrial mutation rate and the rate is sufficiently high in many taxa, making phylogenetic estimates of the mutation rate unreliable.

The direct effects of selection are probably also a minor component of the variation in diversity between species since most analyses attempt to reduce the effects of selection by considering sites that are putatively neutral, such as synonymous sites. Although selection is known to act at synonymous sites in some organisms, this selection appears to be generally weak (Hershberg and Petrov 2008) and will thus have limited effects on genetic diversity. Selection nevertheless might have indirect effects on genetic diversity through background selection (Charlesworth et al. 1993) and genetic hitchhiking (Maynard Smith and Haigh 1974), something we consider below in our discussion of the effective population size.

Since variation in the mutation rate and the direct effects of selection probably have relatively little influence on the variation in diversity between species, population geneticists have focused most attention on factors that might affect the effective population size. It seems logical that the effective population size should be related to the census population size, and levels of genetic diversity are generally related to measures of census population size for nuclear DNA (see compilation of studies by James and Eyre-Walker 2020 and most recent analysis of this problem by Buffalo 2021). However, this relationship is not found consistently for mitochondrial DNA (James and Eyre-Walker 2020). The less consistent picture in mtDNA might be due to several factors. First, mtDNA is subject to a greater level of stochasticity because there is little or no recombination; all sites in the mitochondrial genome therefore share the same genealogy and this will increase the variance in diversity between species. Second, the mitochondrial genome is short, and this increases the level of sampling error. Lastly, it might also be subject to high levels of stochasticity through genetic hitchhiking, since the spread of an advantageous mutation removes all genetic variation from the mitochondrial genome (Bazin et al. 2006).

In those studies in which the relationship between diversity and census population size has been quantified, the slope is considerably less than one for both nuclear (Soule 1976; Nei and Graur 1984; Leffler et al. 2012; Buffalo 2021) and mitochondrial DNA (James and Eyre-Walker 2020); for example, in the largest analysis to date using nuclear DNA data from 172 metazoan animals, Buffalo (2021) found that 10-fold increase in estimated census population size led to a ∼13% increase in diversity. A qualitatively similar slope was found for mtDNA in mammals (James and Eyre-Walker 2020).

The fact that genetic diversity is less variable than census population size between species is known as Lewontin's paradox (Lewontin 1974). The reasons for this scaling are not known but it seems likely that multiple factors are involved (Charlesworth and Jensen 2022). Several explanations have been proposed that are based on demographic or genetic factors. First, if species go through bottlenecks, then diversity might never reach its equilibrium value in species with large population sizes, because of the time it takes for diversity to asymptote—on the order of *N*_e_ (effective population size) generations (Buffalo 2021; Charlesworth and Jensen 2022). Second, species with larger population sizes might tend to fluctuate more in their population size, reducing their effective population size relative to their census population size more substantially than species which have small but stable population sizes (Romiguier et al. 2014). Third, the partial disconnect between diversity and census population size might be due to genetic hitchhiking (Maynard Smith and Haigh 1974; Gillespie 2000). If species with larger census population sizes tend to undergo more adaptive substitutions, then they will undergo more hitchhiking which will reduce the levels of diversity. There is some evidence that species with large population sizes undergo higher rates of adaptive evolution (Gossmann et al. 2012; Nam et al. 2017; Rousselle et al. 2020) (although see Galtier 2016), and Corbet-Dettig et al. (2015) have shown that species with larger census population sizes appear to have a greater reduction in diversity due to effects of selection at linked sites. However, Corbet-Dettig et al. (2015) also estimate that diversity is reduced by linked selection by at most 70%. This therefore does not explain why the slope of the relationship between diversity and census population size is so shallow; this would require the effects of hitchhiking to vary by many orders of magnitude (Coop 2016). Consistent with this, Buffalo (2021) has recently shown that neither background selection nor genetic hitchhiking is capable of explaining why the relationship between diversity and census population size is so shallow.

Finally, diversity might not show as much variation as census population size because of an interaction between two of the processes that determine the level of neutral variation—the mutation rate and the effective population size. Lynch and colleagues (Lynch 2010; Lynch et al. 2016) have argued that species with large *N*_e_ should have lower mutation rates, because in sexual species, mutation rates are always selected to be reduced (Leigh 1973), the efficiency of this selection will depend upon the effective population size, and selection on modifiers of the mutation rate will be more effective in species with large *N*_e_. The per nucleotide mutation rate does indeed appear to be negatively correlated to the effective population size for nuclear and mitochondrial DNA (Lynch 2010; Lynch et al. 2016). However, the slope is shallower than might be expected; mutation rates in multicellular eukaryotes vary by about one order of magnitude whereas effective population sizes vary by several orders.

In an attempt to understand why species vary in their diversity, we have analyzed data from birds, which have received relatively little attention, despite being one of the best characterized groups in terms of their census population sizes and ranges. Previous analyses have suggested that diversity is correlated to census population size; James et al. (2016) showed that mtDNA diversity in island birds is significantly lower than close relatives on the mainland. This is an elegant example of Lewontin's paradox, because the ranges of the island species were on average 1,000-fold smaller than their mainland counterparts, but diversity was reduced by just 60%. These differences between island and mainland species have been corroborated in a more extensive analysis by Leroy et al. (2021), who not only show that island species have lower diversity in their nuclear genome than mainland species, but that DNA diversity is correlated to census population size and range. Furthermore, they show that an expected correlate of the effective population size, the ratio of nonsynonymous to synonymous nucleotide diversity, *π*_N_/*π*_S_, is also correlated to measures of census and effective population size, suggesting that the effective and census population sizes are correlated. However, as with other analyses, the relationship between effective and census population size appears to be shallow. Finally, Figuet et al. (2016) have shown that nuclear DNA sequence diversity is positively correlated to body mass, longevity, and age of sexual maturity in birds, whereas *π*_N_/*π* is negatively correlated to each of these variables. This suggests that each factor is correlated to effective population size and that nuclear diversity depends on the effective population size.

Here, we consider the relationship between diversity in the mitochondrial genome and measures of census population size, as well as other variables that have been shown to correlate to diversity, in birds. We focus our attention on diversity in the mitochondrial genome since it has received very little investigation. We find that the strongest correlate is range size, but confirming Lewontin's paradox, we find that the slope is shallow.

## Results

Using publicly available data, we compiled mitochondrial polymorphism data from over 300 bird species. Between 2 and 420 individuals had been sequenced with a median sample size of 21. We are interested in what factors might affect the level of diversity and hence we compiled estimates of range size, current population size, latitude, adult body mass, longevity, mass-specific metabolic rate (MSMR), egg mass, and clutch size from the literature. These traits were chosen either because they have been shown to be correlated to diversity in other species or because we suspect they might be. In particular, Romiguier et al. (2014) have shown that propagule size is the strongest correlate of nuclear diversity across multicellular animals, a correlation they interpret in terms of *r* and *k* selection. They hypothesize that *r*-selected species, although having large population sizes, undergo frequent bottlenecks, and this depresses their effective population size more than species that are *k* selected. Ideally, we would have compiled data on fledging mass, the equivalent of propagule size in birds, but this is only available for a small number of species. We therefore focused on two variables that might be correlated to *r* and *k* selection, clutch size, and egg mass.

We find significant phylogenetic signal for all of our variables using Pagel's *λ*. This statistic varies from zero under a model in which there is no phylogenetic signal—that is, closely related species are no more similar in trait values than distantly related species—to one, when the trait has evolved through Brownian, and closely related species are more similar to each other. We find that Pagel's *λ* is close to one for all traits, except the two diversity statistics and range, which are nevertheless significantly different from zero (table 1). As a consequence, we used phylogenetically independent contrasts in all analyses (Felsenstein 1985), using the logarithm of each variable.

**Table 1:** Results from a Test of Phylogenetic Inertia Using Pagel's *λ*

Trait (Log Values)	Pagel's *λ*	*P* Value
*π*_S_	0.37	0.001
*π*_N_/*π*_S_	0.16	0.005
Range	0.22	0.005
Mass	1.0	0.001
Population size	0.71	0.001
Mass-specific metabolic rate	1.0	0.001
Longevity	0.66	0.001
Absolute latitude (breeding)	0.68	0.001
Absolute latitude (nonbreeding)	0.68	0.001
Egg mass	1.0	0.001
Clutch size	1.0	0.001

Note.—All variables were log-transformed data. We performed a likelihood ratio test against the hypothesis that there is no phylogenetic signal, that is, *λ* = 0.

**Fig. 1.**
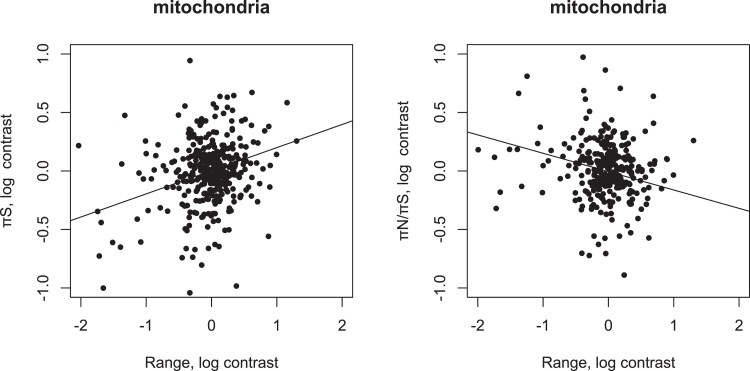
The correlation between geographic range with *π*_S_ (left) and *π*_N_/*π*_S_ (right) in mitochondria DNA: the values plotted are log-transformed phylogenetic contrasts. The regression lines shown have the slope for *π*_S_ of 0.20, *P* < 0.001, and *π*_N_/*π*_S_ of −0.16, *P* < 0.001.

We find that synonymous diversity in the mitochondrial genome is significantly positively correlated to range size (fig. 1) and significantly negatively correlated to clutch size (table 2); these correlations remain significant if we use Spearman's rank correlation (supplementary table S1, Supplementary Material online). Diversity is also significantly correlated to both factors in a multiple regression (*P* < 0.001 for both variables). Either of these correlations could be due to variation in the effective population size or some other factor. To investigate whether the correlations are due to variation in the effective population size, we tested whether either factor was correlated to the ratio of nonsynonymous and synonymous diversities *π*_N_/*π*_S_. The ratio *π*_N_/*π*_S_ is expected to be correlated to the effective population size under the assumption that synonymous mutations are neutral and nonsynonymous mutations are either neutral or deleterious. Under these assumptions, *π*_N_/*π*_S_ is an estimate of the number of nonsynonymous mutations that are effectively neutral and this is expected to be negatively correlated to the effective population size. As expected, we find that *π*_N_/*π*_S_ is negatively correlated to range size (fig. 1), consistent with an increase in effective population size with range size. There is no significant correlation with clutch size, which suggests that this correlation might be driven by other factors, possibly a relationship between clutch size and the mutation rate. We also find that *π*_N_/*π*_S_ is significantly correlated to the absolute latitude of the nonbreeding population, but not that of the breeding population, although this correlation is not significant if we use Spearman's correlation (supplementary table S1, Supplementary Material online).

**Table 2:** The Correlation between *π*_S_ and *π*_N_/*π*_S_ and Various Life History and Demographic Traits

Trait (Log Values)		*n*	Pearson's Correlation Coefficient	
			*r*	*P*
Range	*π*_S_	359	0.39	**<0.001**
	*π*_N_/*π*_S_	316	−0.35	**<0.001**
Absolute latitude (breeding)	*π*_S_	231	−0.064	0.33
	*π*_N_/*π*_S_	201	−0.082	0.25
Absolute latitude (nonbreeding)	*π*_S_	230	0.033	0.62
	*π*_N_/*π*_S_	200	−0.25	**<0.001**
Population size	*π*_S_	101	0.11	0.30
	*π*_N_/*π*_S_	87	−0.17	0.11
Mass	*π*_S_	357	−0.006	0.91
	*π*_N_/*π*_S_	313	0.095	0.095
Mass-specific metabolic rate	*π*_S_	83	−0.046	0.68
	*π*_N_/*π*_S_	76	−0.22	0.054
Longevity	*π*_S_	161	0.12	0.14
	*π*_N_/*π*_S_	143	0.035	0.68
Egg mass	*π*_S_	271	0.026	0.67
	*π*_N_/*π*_S_	238	−0.014	0.83
Clutch size	*π*_S_	350	−0.12	**0.021**
	*π*_N_/*π*_S_	308	−0.015	0.80

Note.—Values were log-transformed before phylogenetic contrasts were calculated. The number of contrasts (*n*) available for each correlation is given, and significant results are in bold.

The relationship between (the contrasts in) log(*π*_S_) and log(range) has a slope of 0.20 (0.03). This slope implies that as range doubles, so diversity increases by ∼15%. For log(*π*_N_/*π*_S_), the slope versus log(range) is −0.16 (0.02) which again is not significantly different to the slope observed in mammals of −0.092 (James and Eyre-Walker 2020).

It is possible that the correlations between *π*_S_, *π*_N_/*π*_S_, and range size might be due to the presence of population structure or cryptic species; if the number of subpopulations or species increases with range size, then *π*_S_ will be positively correlated to range size because *π*_S_ will become dominated by synonymous substitutions between species, and *π*_N_/*π*_S_ may be negatively correlated to range size because *π*_N_/*π*_S_ within species is often greater than the ratio of nonsynonymous and synonymous substitutions between species. To investigate whether population substructure or cryptic species might be a problem, we used methods that model the phylogeny of the sequences as a combination of a neutral coalescent and a Yule process (Reid and Carstens 2012). If the phylogeny is consistent with a neutral coalescent model, then all sequences are attributed to the same species, but if groups of sequences do not fit the coalescent process, then there is evidence of substructure in the data which might indicate separate subpopulations or species. Taking the largest cluster of sequences from each species, which are consistent with being a single panmictic population, we find that *π*_S_ remains positively and *π*_N_/*π*_S_ negatively correlated to range size; all other variables remain uncorrelated with diversity measurements, except for longevity, which becomes significantly negatively correlated with *π*_S_ (supplementary table S2, Supplementary Material online). However, it should be noted that in some species, relatively few individuals have been sequenced and hence we have little power to detect substructure in some of our data.

## Discussion

We have investigated whether genetic diversity in avian mitochondrial genomes is correlated to a number of factors using an extensive data set from over 300 species. We find that diversity at synonymous sites is significantly correlated to range. The correlation between *π*_S_ and range seems to be due to variation in effective population size, since a measure of *N*_e_, the ratio of nonsynonymous to synonymous diversity, *π*_N_/*π*_S_, is significantly negatively correlated to range size. This is as we might expect since census population size is likely to be correlated to range, and the effective population size should be correlated to census population size. The slope of the relationship between diversity and range is shallow, consistent with Lewontin's paradox; it is such that as range doubles, diversity increases by just 15%. The correlation is also such that log(range) explains only 15% of the variance in diversity. Perhaps surprisingly, we find no correlation between the current population size and either *π*_S_ or *π*_N_/*π*_S_. This might be due to the small sample size that we have for current population size estimates and the large variance associated with these estimates.

A number of studies do not show a correlation between mitochondrial diversity and census population size (see table s2 from James and Eyre-Walker 2020). It is difficult to know whether this reflects statistical power or fundamental differences between groups of organisms. Statistical power does often appear to be a problem; early studies in both mammals (Nabholz et al. 2008, 2009) and birds (Nabholz et al. 2009) failed to find a significant correlation between mitochondrial diversity and measures of census population size, but subsequent studies have revealed these relationships with larger data sets (James and Eyre-Walker 2020 and this study). This is perhaps not surprising given how little of the variance in diversity range size explains; there is a considerable amount of variance that is left unexplained by measures of census population size, and hence, this correlation is difficult to detect without large sample sizes.

We have assumed that census population is correlated to range size, and for our data, this correlation is confirmed [log(census population size) is significantly correlated to log(range size)] (*r* = 0.79, *P* < 0.001). This correlation is surprisingly strong given that we have not taken into account population density, which might be just as important in determining population size as range. Population density is weakly negatively correlated to body size in birds (Juanes 1986), probably reflecting the simple principle that larger animals have bigger energy budgets and hence need a larger area for each individual. However, population size is not more strongly correlated to range divided by mass in our data (*r* = 0.77, *P* < 0.001), than simply range (*r* = 0.79, *P* < 0.001).

It is striking that the slope of the relationship between log(*π*_S_) and log(range) in birds [*b* = 0.20 (0.03)] is similar, and not significantly different, to the slope observed for mammalian mitochondrial DNA of 0.16 (James and Eyre-Walker 2020). The similarity of the patterns in birds and mammals is perhaps surprising because in birds, the mtDNA is linked to the W chromosome, which may be subject to the Hill–Robertson effects (Berlin et al. 2007). Such effects might be expected to depend on population size and hence affect the relationship between effective and census population sizes.

The slopes of the relationship between diversity and census population size in birds and mammals are however considerably steeper than the slope observed for nuclear DNA across 172 metazoan taxa, which is 0.06 (Buffalo 2021). The reason for this difference is not clear but surprisingly it suggests that mitochondrial DNA varies more as a function of census population size than nuclear diversity. This is unexpected given that the relationship between diversity and census population size has been found in many more studies of nuclear than mitochondrial DNA (James and Eyre-Walker 2020).

There are few other significant correlations in our analysis. Synonymous diversity is positively correlated to clutch size consistent with the hypothesis that *r* strategy species have higher diversity than *k* strategists (Romiguier et al. 2014). However, there is no evidence that this relationship is associated with effective population size, because *π*_N_/*π*_S_ is not correlated to clutch size. It is possible that species with large clutch size have higher mutation rates, and as a consequence, higher diversity, than species with small clutch size. However, birds are probably not a good system to test hypotheses about *r* and *k* selection since all birds invest in parental care and there is relatively little variance in reproductive output between species. The correlation between diversity and propagule size was found when comparing species across the diversity of multicellular animals, with propagule size and reproductive output, in terms of offspring, varying by many orders of magnitude (Romiguier et al. 2014).

Unfortunately, our analysis does not yield any insight into the reasons for Lewontin's paradox. There are currently many hypotheses for why nucleotide diversity might not scale with census population size and it is likely that there is no single reason (Charlesworth and Jensen 2022).

## Materials and Methods

Mitochondria and nuclear sequences were downloaded from NCBI PopSet. We included all species for which at least two individuals had been sequenced. Alignment of the sequences was performed using Geneious version 7.0.6 (Kearse et al. 2012). Sequences were concatenated if multiple genes had been sequenced from the same individual. If multiple genes had been sequenced from different individuals, then the mean values of *π*_S_ and *π*_N_/*π*_S_ were calculated. Synonymous and nonsynonymous diversity were calculated using in-house scripts. All variables were analyzed on a log scale so *π*_N_ and *π*_S_ are undefined if a species has no nonsynonymous or synonymous diversity; such species were excluded from the analysis.

Life history trait information and geographic distribution maps were downloaded from Bird Life International (www.birdlife.org; last accessed May 6, 2019). We compiled data on seven life history traits: geographic range (in km^2^); median latitude of geographic range, in absolute value representing distance from the equator; adult body mass (in g); maximum longevity (in months); MSMR, calculated by dividing basal metabolic rate (measured in ml O^2^/h) by body mass (in g); clutch size; and egg mass. To calculate the absolute latitude, we took the range maps supplied by Bird Life International and calculated the centroid of the range.

It is important in comparative analyses to control for phylogenetic effects, if they exist. We tested for phylogenetic inertia using Pagel's *λ* (reviewed in Freckleton et al. 2002; Kamilar and Cooper 2013), calculated with the R package phylosignal (Keck et al. 2016). Since all variables showed significant phylogenetic effects, we controlled for these using the method of independent contrasts (Felsenstein 1985). Life history and molecular evolution traits were first log transformed, and then phylogenetic contrasts were calculated using the *ape* package in R (Paradis et al. 2004). We used TimeTree to create the phylogenetic trees used in this study (Hedges et al. 2006; Kumar et al. 2022).

We investigated whether there was population structure or subspecies within the data from each species using a generalized mixed Yule coalescent model (Reid and Carstens 2012). In this model, the times between successive nodes on a phylogenetic tree are modelled as a combination of a Yule process, representing speciation, and a neutral coalescence process, representing diversification between lineages within a species (Reid and Carstens 2012). For each mitochondrial DNA data set (i.e., a set of mitochondrial sequences from a single named species), we estimated the phylogenetic tree using BEAST2.5 (version 2.6.3) (Bouckaert et al. 2019), with a coalescent constant size tree prior and an HKY substitution model assuming all branches on the tree have the same rate of evolution. We ran each analysis for 10 million generations, sampling every 1,000 generations, and assessed the convergence of the Monte Carlo chains and the effectiveness of the burn-in period by examining the effective sample size (ESS) values and likelihood plots in Tracer v.1.5 (Bouckaert et al. 2019). From the sample of trees, we constructed the consensus using Tree Annotator from BEAST2.5 (Bouckaert et al. 2019). To investigate whether there was substructure within this tree, that is, whether the tree was consistent with a neutral coalescent model, we used a generalized mixed Yule coalescent model implemented in the R package *splits* (Ezard et al. 2009) using a single threshold of 0.90. We recorded the most likely number of clusters within given species. For species that produce multiple clusters, we chose the cluster with the most individuals and reestimated *π*_N_ and *π*_S_. All the subsequence analyses were carried out as described.

## Supplementary Material

evad064_Supplementary_Data

## Data Availability

The alignments, estimates of nucleotide diversity, and all other variables used in this study can be obtained at https://github.com/AdamEyreWalker/bird_mtDNA_diversity.
