# Supplementary material for: What Determines Levels of Mitochondrial Genetic Diversity in Birds?
Source: Genome Biol Evol. 2023 Apr 25;15(5):evad064. doi: 10.1093/gbe/evad064 (PMC10159584; doi:10.1093/gbe/evad064)
Supplement: evad064_Supplementary_Data [file evad064_supplementary_data.docx]

**Supplementary information**

| *Trait (Log values)* |  | *n* | Pearson’s correlation coefficient | | Spearman’s ranks correlation coefficient | |
| --- | --- | --- | --- | --- | --- | --- |
|  |  |  | R | *p* | rho | *p* |
| Range | *π_S_* | 359 | 0.387 | **3.25E-14** | 0.151 | **0.004** |
|  | *π_N_*/*π_S_* | 316 | -0.348 | **2.10E-10** | -0.109 | **0.053** |
| Absolute latitude (breeding) | *π_S_* | 231 | -0.064 | 0.334 | -0.113 | 0.087 |
|  | *π_N_*/*π_S_* | 201 | -0.082 | 0.250 | -0.030 | 0.671 |
| Absolute latitude (non-breeding) | *π_S_* | 230 | 0.033 | 0.621 | -0.056 | 0.401 |
|  | *π_N_*/*π_S_* | 200 | -0.245 | **4.91E-4** | -0.090 | 0.205 |
| Population size | *π_S_* | 101 | 0.106 | 0.295 | 0.156 | 0.121 |
|  | *π_N_*/*π_S_* | 87 | -0.173 | 0.111 | -0.147 | 0.176 |
| Mass | *π_S_* | 357 | -0.006 | 0.909 | -0.043 | 0.423 |
|  | *π_N_*/*π_S_* | 313 | 0.095 | 0.095 | **0.113** | **0.047** |
| Mass-specific metabolic rate | *π_S_* | 83 | -0.046 | 0.683 | -0.095 | 0.395 |
|  | *π_N_*/*π_S_* | 76 | -0.224 | 0.054 | -0.137 | 0.240 |
| Longevity | *π_S_* | 161 | 0.117 | 0.141 | 0.055 | 0.488 |
|  | *π_N_*/*π_S_* | 143 | 0.035 | 0.678 | 0.065 | 0.444 |
| Egg mass | *π_S_* | 271 | 0.026 | 0.674 | 0.018 | 0.766 |
|  | *π_N_*/*π_S_* | 238 | -0.014 | 0.831 | -0.018 | 0.780 |
| Clutch size | *π_S_* | 350 | -0.123 | **0.021** | -0.116 | **0.030** |
|  | *π_N_*/*π_S_* | 308 | -0.015 | 0.798 | 0.004 | 0.949 |

**Table S1**. The Pearson’s and Spearman’s correlations between *π_S_*, *π_N_*/*π_S_* and various life history and demographic traits. Values are log-transformed before phylogenetic contrasts are calculated. The column *n* gives the number of contrasts available for each correlation. Significant results are in bold.

| *Trait (Log values)* |  | *n* | Pearson’s correlation coefficient | | Spearman’s ranks correlation coefficient | |
| --- | --- | --- | --- | --- | --- | --- |
|  |  |  | R | *p* | rho | *p* |
| Range | *π_S_* | 333 | 0.298 | **3.04E-08** | 0.113 | **0.038** |
|  | *π_N_*/*π_S_* | 250 | -0.355 | **8.05E-09** | -0.093 | 0.144 |
| Absolute latitude (breeding) | *π_S_* | 211 | 0.020 | 0.769 | -0.116 | 0.095 |
|  | *π_N_*/*π_S_* | 156 | -0.036 | 0.659 | -0.016 | 0.843 |
| Absolute latitude (non-breeding) | *π_S_* | 230 | 0.033 | 0.621 | -0.056 | 0.401 |
|  | *π_N_*/*π_S_* | 155 | -0.141 | 0.082 | -0.115 | 0.157 |
| Population size | *π_S_* | 92 | -0.035 | 0.741 | 0.023 | 0.832 |
|  | *π_N_*/*π_S_* | 87 | -0.173 | 0.111 | -0.147 | 0.176 |
| Mass | *π_S_* | 331 | -0.013 | 0.820 | -0.036 | 0.511 |
|  | *π_N_*/*π_S_* | 247 | 0.052 | 0.418 | 0.017 | 0.796 |
| Mass-specific metabolic rate | *π_S_* | 76 | 0.116 | 0.322 | 0.146 | 0.211 |
|  | *π_N_*/*π_S_* | 59 | -0.192 | 0.149 | -0.178 | 0.180 |
| Longevity | *π_S_* | 147 | -0.248 | 0.003 | -0.199 | 0.016 |
|  | *π_N_*/*π_S_* | 112 | -0.044 | 0.644 | 0.154 | 0.106 |
| Egg mass | *π_S_* | 251 | 0.013 | 0.838 | -0.019 | 0.767 |
|  | *π_N_*/*π_S_* | 188 | 0.026 | 0.719 | 0.004 | 0.958 |
| Clutch size | *π_S_* | 325 | -0.084 | 0.132 | -0.092 | 0.099 |
|  | *π_N_*/*π_S_* | 246 | -0.042 | 0.511 | -0.010 | 0.871 |

**Table S2**. The correlation between *π_S_*, *π_N_*/*π_S_* and various life history and demographic traits for the largest group of sequences within each names species that are consistent with the samples coming from a single panmictic population. Values are log-transformed before phylogenetic contrasts are calculated. The column *n* gives the number of contrasts available for each correlation. Significant results are in bold.
